# Genetic deletion of calcium/calmodulin-dependent protein kinase kinase β (CaMKK β) or CaMK IV exacerbates stroke outcomes in ovariectomized (OVXed) female mice

**DOI:** 10.1186/s12868-014-0118-2

**Published:** 2014-10-21

**Authors:** Lin Liu, Louise McCullough, Jun Li

**Affiliations:** Department of Neuroscience, University of Connecticut Health Center, 263 Farmington Avenue, MC3401, Farmington, CT 06030 USA; Department of Neurology, University of Connecticut Health Center, Farmington, CT USA

**Keywords:** Stroke, CaMKK β, CaMK IV, Inflammation, Sex differences

## Abstract

**Background:**

Stroke is the primary cause of long-term disability in the United States. Interestingly, mounting evidence has suggested potential sex differences in the response to stroke treatment in patients as, at least in part, distinct cell death programs may be triggered in females and males following stroke. The NIH has recognized that females are strikingly under-represented in pre-clinical trials. Calcium/calmodulin-dependent protein kinase kinase (CaMKK) is a major kinase that is activated by elevated intracellular calcium. It has recently been suggested that CaMKK and CaMK IV, a downstream target molecule, are neuroprotective in stroke in males. In this study, we examined stroke outcomes in ovariectomized CaMKK β and CaMK IV deficient females. Cell death/survival signaling and inflammatory responses were assessed.

**Results:**

Our results demonstrated that CaMKK β or CaMK IV KO exacerbated both ischemic injury and behavioral deficits in female mice. Genetic deletion of CaMKK β or CaMK IV increased hemorrhagic transformation after stroke, and this was associated with both increased MMP9 activity and loss of the blood brain barrier (BBB) protein collagen IV. Transcriptional inactivation was observed in mice lacking either CaMKK β or CaMK IV, as indicated by reduced levels of phosphorylated cAMP response element-binding protein (p-CREB) and B-cell lymphoma 2 (BCL-2) proteins. Finally, inhibiting this pathway exacerbated the inflammatory response to stroke as CaMKK β or CaMK IV KO mice had increased levels of the pro-inflammatory serum cytokines tumor necrosis factor alpha (TNFα) and interleukin 6 (IL-6) after stroke. This suggests that the CaMKK pathway is involved in the immune response to brain injury.

**Conclusions:**

Inhibition of CaMKK signaling exacerbated stroke outcome and increased BBB impairment, transcriptional inactivation and inflammatory responses in females after stroke. Therefore, CaMKK signaling may be a potential target for stroke treatment in both males and females.

**Electronic supplementary material:**

The online version of this article (doi:10.1186/s12868-014-0118-2) contains supplementary material, which is available to authorized users.

## Background

Stroke is the primary cause of long-term disability in the United States [1]. Despite decades of research, the only currently available treatment for stroke is t-PA, which has a very narrow therapeutic window owing to the risk of hemorrhage [2]. The epidemiology of human stroke is sexually dimorphic, and stroke rates are higher in men than in women until advanced ages [3]. This sexual dimorphism is thought to result in part from differences in gonadal hormone levels, particularly estrogen. However, the effect of biological sex on stroke outcome is evident even in clinical populations with only minimal differences in hormone levels, such as neonates and the elderly, which suggests that non-hormonal factors also contribute to outcome [4]. Indeed, experimental stroke studies have demonstrated that distinct cell death programs are triggered in females and males following stroke. For example, poly-ADP-ribose polymerase (PARP) is thought to be the dominant pathway for neuronal death in males, whereas in females, caspase-dependent cell death is the dominant pathway after stroke [5]. Based on this mounting evidence supporting potential sex differences in responses to stroke treatment, the criteria developed by the Stroke Therapy Academic Industry Roundtable (STAIR) urge scientists to also study the responses of female animals to experimental stroke [6]. Additionally beginning in October 2014, NIH will require grants applicants to report their plans for the balance of male and female cells and animals in preclinical studies in future applications [7].

Calcium/calmodulin-dependent protein kinase kinase (CaMKK) is a major kinase that is activated by elevated intracellular calcium and is highly abundant in the brain [8]. Upon activation, CaMKK phosphorylates its downstream substrates CaMK I/IV. We have previously shown that inhibiting the CaMKK/CaMK IV pathway exacerbates infarcts and behavioral deficits after cerebral ischemia in male mice, which suggests that this pathway is endogenously protective in stroke [9]. The underlying mechanisms are not fully known, but they may involve increased blood brain barrier (BBB) protection and regulation of transcriptional activation. Interestingly, studies have suggested that the CaMKK pathway has functionally different effects in hippocampal memory formation in male and female mice. In males, CaMKK β (one of the two CaMKK isoforms) is required for spatial memory formation, and deleting this gene leads to impairment in long-term potentiation at hippocampal CA1 synapses. However, this deficit was not observed in female mice [10]. This male-specific function of CaMKK in memory formation may be due to gene transcription that only occurs in males [10]. Because transcriptional regulation is thought to be a key mechanism by which CaMKK achieves its neuroprotective role in stroke [9], the role of CaMKK signaling in response to stroke may differ between the sexes. Therefore, we investigated the effects of deleting both CaMKK β and one of its two downstream targets, CaMK IV in female mice after stroke.

## Methods

### Animals

All experiments were approved by the Center for Laboratory Animal Care of the University of Connecticut Health Center and were performed in accordance with the National Institutes of Health guidelines for the care and use of laboratory animals. Both the CaMKK β knockout (KO) mice and the CaMK IV KO mice were provided by Dr. Anthony Means at Duke University and were backcrossed into the C57BL/6 J background for at least 10 generations. Both KO strains are normal in size and do not display any gross physical or behavioral abnormalities. F1 heterozygous mattings were used to generate control female mice, which were subsequently backcrossed into the C57BL/6 J background. Female mice that were age- and weight-matched were used in all experiments (20–24 g, 10–12 weeks of age). All of the mice were OVXed 10 days before undergoing middle cerebral artery occlusion (MCAO) as previously described [11]. We used OVXed animals in this study to control for the levels of gonadal hormones and to reduce the experimental variability that is associated with varying estrogen levels during estrus. This is also a more appropriate model for the “at risk” clinical population because the vast majority of women who experience stroke are postmenopausal [4].

### Middle cerebral artery occlusion

We induced focal transient cerebral ischemia (90 minutes of MCAO) in CaMKK β or CaMK IV KO and WT female (control) mice using previously established methods [12]. After 90 minutes of occlusion, the mice were re-anesthetized and re-perfused by suture withdrawal. During the ischemic period, their body temperature was controlled at a physiological level using a heating pad with a feedback thermo-control system (FST). The animals were randomized into the stroke and surgical sham cohorts. Mice in the sham group underwent the same procedure except that the MCA was not occluded.

### Behavior measurement

At 72 hours after the onset of stroke, neurological deficits were scored on a four-point scale as previously described, where 0: no deficit; 1: forelimb weakness and torso turning to the ipsilateral side when held by the tail; 2: circling to the affected side; 3: unable to bear weight on the affected side; and 4: no spontaneous locomotor activity or barrel rolling [12].

### Hemoglobin assay

At 72 hours after the onset of stroke, mice were anesthetized with Avertin and perfused transcardially with cold PBS containing heparin (100 U/mL). The brains were removed, sliced, and initially used for 2,3,5-triphenyl-2 h-tetrazolium chloride (TTC) staining as described below. The brain slices were then dissected to separate the affected and unaffected hemispheres, and quantification of hemorrhagic transformation (HT) was performed using a spectrophotometric hemoglobin assay as previously described [12]. The affected hemispheres were homogenized by sonication in distilled water, followed by centrifugation at 13,000 rpm for 30 minutes. After the supernatant was collected, 200 μl of reaction reagent (QuantiChrom Hemoglobin Assay Kit; BioAssay Systems, Hayward, CA, USA) was added to each 50 μl of supernatant, and the samples were allowed to stand for 15 minutes before the optical density was measured at 405 nm using a spectrophotometer (Wallace 1420, PerkinElmer, Waltham, MA, USA). Finally, the total hemoglobin concentration was calculated as micrograms per hemisphere [12].

### Infarct measurement

At 72 hours after the onset of stroke, the animals were sacrificed, and the brains were immediately removed and cut into 5 individual 2-mm slices. These brain slices were stained with 1.5% 2, 3, 5-triphenyltetrazolium (TTC) at 37°C for 8 minutes. Sigmascan Pro5 software was used to analyze infarct volumes (corrected for edema) after the brain TTC images were digitized, as previously described [13]. Using the same software, edema formation (brain swelling) was calculated. Edema = 100% * (ipsilateral hemisphere volume - contralateral hemisphere volume)/ contralateral hemisphere volume.

### Immunoblotting

To detect protein levels, mouse brains were harvested 6 hours after the onset of stroke and then homogenized in RIPA lysis buffer (Boston Bioproducts). The concentration of protein in each sample was determined with a BCA assay (Thermo Scientific). The proteins were separated using precast gels (7.5%, 10%, 12% or 4-15%, Bio-Rad) and were then transferred to polyvinylidene difluoride membranes (Bio-Rad). After the transfer and blocking steps, the blots were incubated overnight at 4°C with primary antibodies (anti-Collagen IV antibody, 1:500, Abcam; anti-p-CREB antibody, 1:1000, Abcam; or anti-BCL-2 antibody, 1:1000, Cell Signaling Technology) diluted in Tris-buffered saline containing 0.1% Tween-20 and 4% bovine serum albumin or 5% fat free milk. The appropriate secondary antibodies (anti-rabbit IgG, 1:5000, Cell Signaling Technology; or anti-mouse IgG, 1:5000, Vector) were diluted in the same blocking buffer, and an electrochemiluminescence detection kit (Thermo Scientific) was used for signal detection. β-actin (primary antibody 1:5000; Sigma) was used as a loading control.

### Gelatin zymography

We employed gelatin zymography to assess the activity of matrix metalloproteinase-9 (MMP-9). At 6 hours after stroke, the mice were sacrificed, and their brains were removed and homogenized using RIPA lysis buffer (Cell signaling). Aliquots of the supernatant, each containing 300 μg of protein, were subjected to affinity precipitation with gelatin-conjugated sepharose beads (GE, Life Science). The bound material was released from the beads in 50 μl of elution buffer with 10% DMSO. Finally, the samples were analyzed with 10% gelatin zymogram gel (Bio-Rad) [9].

### Enzyme-linked immunosorbent assay (ELISA)

Blood samples were collected transcardially at 72 hours after stroke and were spun at 6000 r.p.m. for 10 minutes at 4°C. Cytokines were detected by ELISA, performed according to the manufacturer’s instructions (eBioscience, US) [12].

### Statistical analysis

All data are presented as the mean ± SEM except for neurological scores, which are expressed as the median (IQR). One-way ANOVA was used to perform mean comparisons between the experimental groups, and a post-hoc test (Tukey’s) was then used for multiple comparisons. Neurological scores were compared with the Mann–Whitney *U* test. A value of p < 0.05 was considered statistically significant. Behavioral and histological assessments were conducted by an investigator who was blinded to the genotype/drug treatment.

## Results

### CaMKK β and CaMK IV protein levels did not differ between male and females

**Figure 1:**
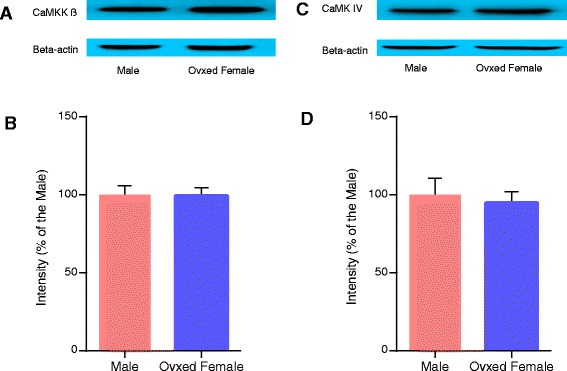
**Baseline levels of CaMKK β and CaMK IV in males and females. A and C**, representative of CaMKK β and CaMK IV by Western blot; **B and D**, quantification of CaMKK β and CaMK IV levels, n = 6 for each group. Data were normalized to corresponding males controls and presented as mean ± SEM. Ovxed: ovariectomized.

We used Western blots to measure protein levels of CaMKK β and CaMK IV at baseline in both males and females. Female ovarectomized mice and male mice displayed equal CaMKK β and CaMK IV protein levels in the brain (n = 6 for each group) (Figure 1).

### CaMKK β knockout exacerbated stroke injury

**Figure 2:**
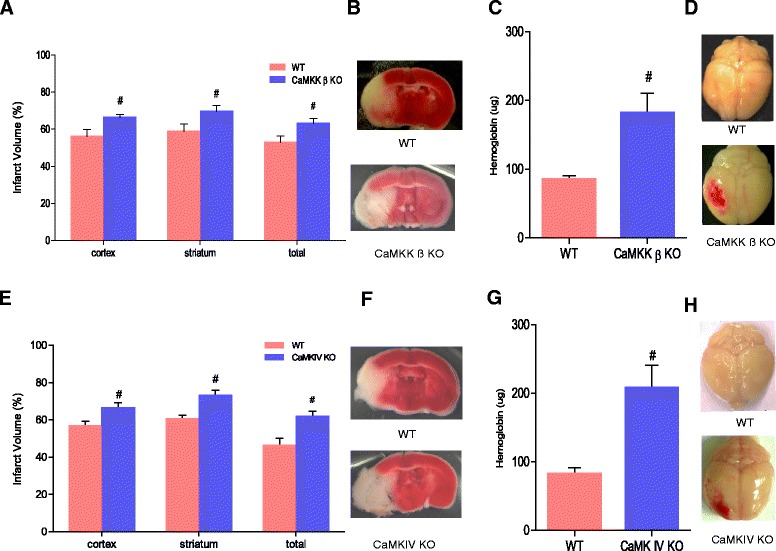
**Loss of CaMKK β/CaMK IV exacerbated stroke infarct volumes and cerebral hemorrhagic transformation (HT) in females. A and E**, Infarct volumes in CaMKK β/CaMK IV KO females mice; **C and G**, Hemoglobin content were higher in CaMKK β/CaMK IV KO females 72 hours after stroke; concentration unit: ug/per hemisphere; **B and F**, representative brains with cerebral infarcts in CaMKK β/CaMK IV KO females and corresponding controls; **D and H**, representative brains with cerebral HT in CaMKK β/CaMK IV KO females and corresponding controls; All female mice were ovxed 10 days before MCAO. In figure A and C, n = 7 WT/ n = 9 KO, in figure D and G, n = 8 for each group; ^#^p < 0.05: versus WT controls. Student *t*-test was used to compare means. Data were presented as mean ± SEM.

At 72 hours after stroke onset, CaMKK β KO OVXed female mice had significantly larger infarcts in the cortex, the striatum and the total hemisphere compared to WT controls (cortex: KO 66.1 ± 1.7% versus WT 55.9 ± 3.9%, p < 0.05; striatum: KO 69.6 ± 3.1% versus WT 58.7 ± 4.0%, p < 0.05; total: KO 63.1 ± 2.7% versus WT 52.6 ± 3.7%, p < 0.05, n = 7 WT and n = 9 KO) (Figure 2A and B). Exacerbated hemispheric swelling was observed in CaMKK β KO females (10.82 ± 1.94% in WT versus 16.74 ± 1.59% in CaMKK β KO, n = 7 WT and n = 9 KO) (Additional file 1: Figure S1A). These larger infarcts and exacerbated hemispheric swelling contributed to increased neurological deficit scores, demonstrating a worse functional outcome (Additional file 2: Table S1). Furthermore, knockout of the CaMKK β gene increased hemorrhagic transformation as measured by a hemoglobin assay 72 hours after stroke (85.0 ± 5.5 μg/hemisphere in the WT (n = 7) versus 181.7 ± 28.7 μg/hemisphere in the KO (n = 9), p < 0.05) (Figure 2C and D). No difference was observed in mortality rates between CaMKK β KO and WT mice after stroke.

To confirm that gonadal estrogen was reduced, we weighed the uterus 13 days after the mice underwent ovariectomy. Uterine weights were significantly decreased in both OVXed WT and OVXed CaMKK β KO mice (25.5 ± 1.9 mg for OVXed WT (n = 7) versus 96.7 ± 20.0 mg for WT sham (n = 5), p < 0.05; 26.2 ± 1.7 mg for OVXed CaMKK β KO (n = 9) versus 117.5 ± 16.6 mg for CaMKK β KO sham (n = 5), p < 0.05).

### CaMK IV knockout worsened stroke outcome

Knockout of CaMK IV significantly increased the size of infarcts in the cortex, the striatum and total hemisphere at 72 hours after stroke onset (cortex: 66.1 ± 3.1% in the KO versus 56.5 ± 2.6% in the WT, p < 0.05, striatum: 72.6 ± 3.4% in the KO versus 60.0 ± 2.5% in the WT, p < 0.05, total: 61.6 ± 3.0% in the KO versus 46.1 ± 4.0% in the WT p < 0.05, n = 8 for each group) (Figure 2E and F). Exacerbated hemispheric swelling was observed in CaMK IV KO females (11.24 ± 1.68% in WT versus 17.29 ± 2.41% in CaMK IV KO, n = 8 for each group) (Additional file 1: Figure S1B). Additionally, CaMK IV KO mice performed worse on neurological deficit tests (Additional file 2: Table S1). Furthermore, the loss of CaMK IV significantly increased hemorrhagic transformation as evaluated by a hemoglobin assay 72 hours after stroke (82.5 ± 8.6 μg/hemisphere in the WT versus 207.3 ± 33.2 μg/hemisphere in the CaMK IV KO, p < 0.05, n = 8 for each group) (Figure 2G and H). This result was similar to our previous observation in CaMKK β KO mice. No difference in mortality rate was observed between CaMK IV KO and WT mice after stroke.

Similarly, uterine weights of OVXed CaMK IV KO mice and the corresponding OVXed WT controls were also significantly decreased compared to mice with intact ovaries (22.8 ± 1.9 mg for OVXed WT (n = 8) versus 102.4 ± 23.2 mg for WT sham (n = 5), p < 0.05; 23.7 ± 1.9 mg for OVXed CaMK IV KO (n = 8) versus 106.3 ± 16.8 mg for CaMK IV KO sham (n = 5), p < 0.05).

### Loss of CaMKK β or CaMK IV increased gelatinase activity and reduced collagen IV levels 6 hours after stroke

**Figure 3:**
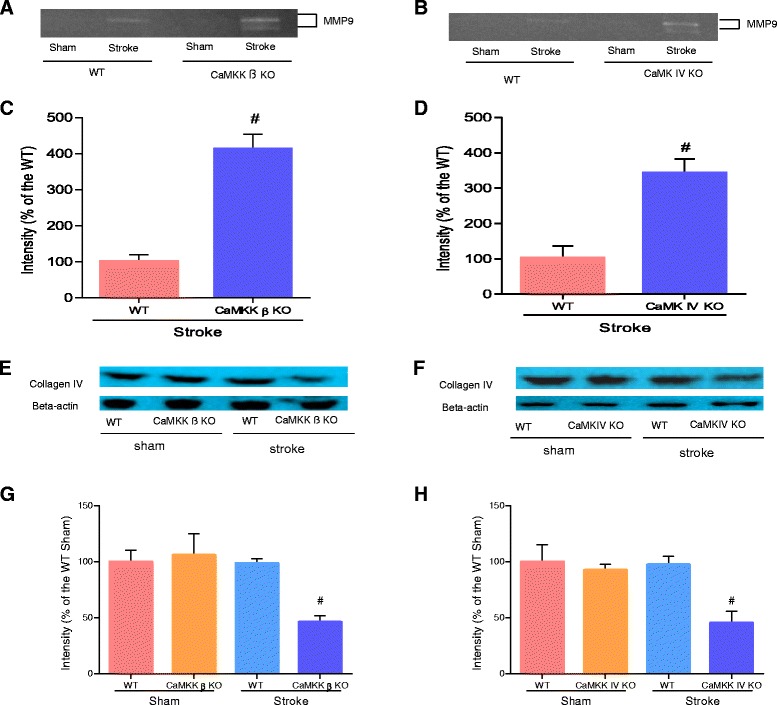
**Loss of CaMKK β or CaMK IV increased gelatinase activity and reduced collagen IV levels 6 hours after stroke. A and B**, representative bands of MMP-9 expression assessed by gelatin zymograpy in CaMKK β/CaMK IV KO mice. **C and D**, quantification of MMP-9 expression, n = 3 for each group. Data were normalized to corresponding WT stroke mice in each strain. **E and F**, representative of collagen IV by Western blot in CaMKK β/CaMK IV KO mice. **G and H**, quantification of collagen IV expression, n = 3 sham/n = 4 stroke. Data were normalized to corresponding WT sham. ^#^P < 0.05 compared to WT stroke; Data were presented as mean ± SEM.

To explore the mechanisms underlying the increased HT in both CaMKK β KO and CaMK IV KO mice, we examined the activity of matrix metalloproteinases (MMPs) after stroke onset. Loss of CaMKK β resulted in significantly upregulated MMP-9 levels 6 hours after stroke (Figure 3A and C, n = 3 per stroke group, p < 0.05). Similar results were obtained with CaMK IV KO mice (Figure 3B and D, n = 3 per stroke group, p < 0.05). Furthermore, we used Western blotting of samples from 6 hours after stroke onset to quantify the levels of collagen IV protein, which is an important component of the BBB and a substrate of MMPs. Collagen IV levels were decreased in both KO strains after stroke (Figure 3E-H, n = 3 for the sham group, n = 4 per stroke group). Additionally, in the sham groups, we observed no difference between the KO animals and their corresponding WT controls, implying that the loss of either CaMKK β or CaMK IV did not affect the baseline levels of BBB integrity (Figure 3E-H, n = 3 for the sham group, n = 4 for each stroke group).

### Genetic deletion of CaMKK β or CaMK IV reduced p-CREB and BCL-2 levels 6 hours after stroke

**Figure 4:**
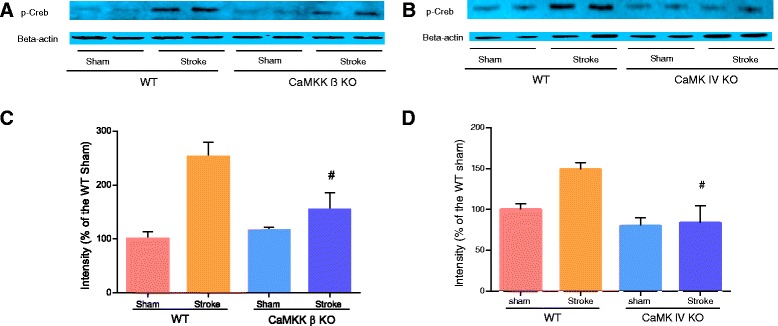
**Loss of CaMKK β or CaMK IV reduced p-CREB levels 6 hours after stroke. A and B**, representative Western blots of p-CREB in CaMKK β/CaMK IV KO mice. **C and D**, quantification of p-CREB expression, n = 2 sham/n = 3 stroke. Data were normalized to corresponding WT sham. ^#^P < 0.05 compared to WT stroke; Data were presented as mean ± SEM.

**Figure 5:**
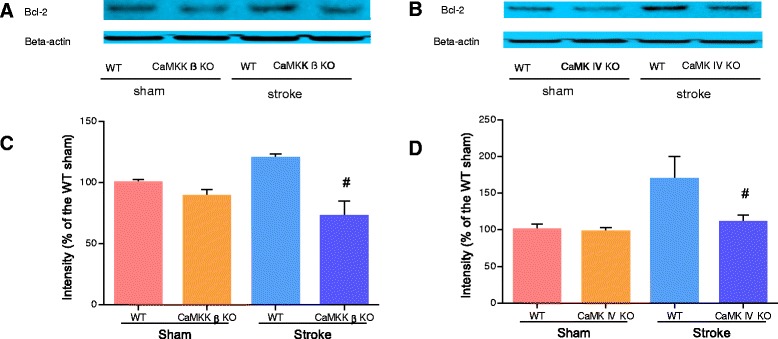
**Loss of CaMKK β/CaMK IV decreased Bcl-2 levels 6 hours after stroke. A and B**, representative Western blots of Bcl-2 in CaMKK β/CaMK IV KO mice. **C and D**, quantification of Bcl-2 expression, n = 2 WT/n = 3 KO. Data were normalized to corresponding WT sham. ^#^P < 0.05 compared to WT stroke; Data were presented as mean ± SEM.

Next, we examined the levels of p-CREB, which is a neuroprotective transcription factor that is activated by CaMK IV [14]. At 6 hours after stroke, p-CREB levels were significantly reduced in CaMKK β KO mice when compared to their corresponding WT controls (Figure 4A and C; n = 2 for the sham group; n = 3 for the stroke group). A similar reduction in p-CREB levels was observed in CaMK IV KO mice 6 hours after stroke (Figure 4B and D; n = 2 for the sham group; n = 3 for the stroke group). BCL-2, which is a downstream target of CREB, has previously been shown to be beneficial in stroke. We therefore studied BCL-2 levels in both CaMKK β KO and CaMK IV KO mice. Consistent with the p-CREB data, we observed that at 6 hours after stroke, BCL-2 expression was significantly reduced in both KO strains when compared to their corresponding WT controls (Figure 5A-D, n = 2 for the WT group; n = 3 for each KO group).

### Loss of CaMKK β or CaMK IV aggravated the inflammatory response after stroke

**Figure 6:**
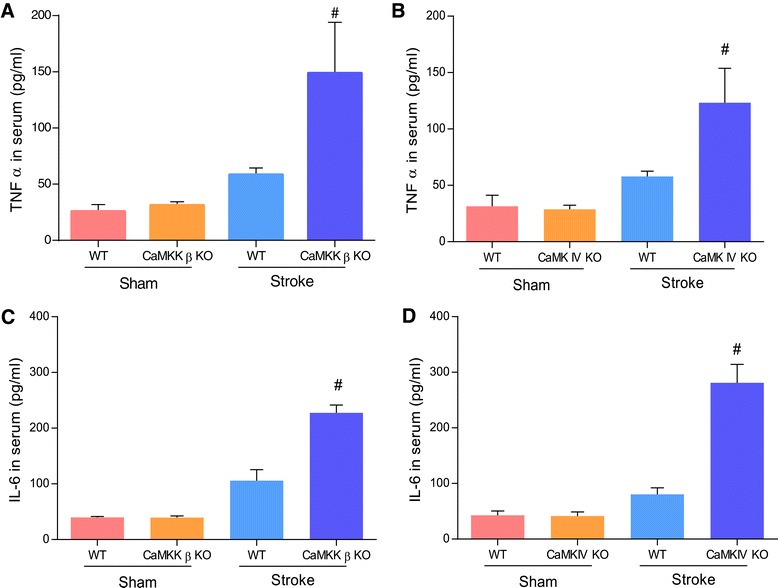
**Loss of CaMKK β or CaMK IV aggravated the inflammatory response after stroke.** TNFα **(A and B)** and IL-6 **(C and D)** was analyzed with ELISA kits. n = 3 for each group. ^#^P < 0.05 compared to WT controls; Data were presented as mean ± SEM.

CaMKK signaling has been implicated in immune regulation, which plays a pivotal role in determining stroke outcome. Therefore, we measured serum levels of IL-6 and TNFα in CaMKK β and CaMK IV KO mice 72 hours after stroke. The loss of either CaMKK β or CaMK IV aggravated the inflammatory response; this was indicated by increased levels of the pro-inflammatory cytokines TNFα (Figure 6A and B) and IL-6 (Figure 6C and D) (n = 3 for each group).

## Discussion

In this study, we utilized OVXed female mice, a translationally relevant model system, to explore the role of the CaMKK/CaMK IV pathway in stroke. First, we demonstrated that levels of CaMKK β and CaMK IV proteins in the female brain were equivalent to that in the males. Second, we found that the genetic deletion of CaMKK β or CaMK IV aggravated both infarcts and behavioral deficits. Third, both CaMKK β and CaMK IV KO mice exhibited increased hemorrhagic transformation after stroke, and this was associated with both increased MMP9 activity and the loss of the BBB protein collagen IV. Fourth, transcriptional inactivation was observed in mice lacking either CaMKK β or CaMK IV, as indicated by the reduced levels of p-CREB and BCL-2. Finally, we demonstrated that the levels of the pro-inflammatory serum cytokines TNFα and IL-6 increased after stroke in both CaMKK β and CaMK IV KO mice, indicating that inhibiting this pathway exacerbates the inflammatory response.

Our data demonstrate that CaMKK signaling may be an endogenous protective mechanism in females even though calcium signaling has traditionally been thought to play a detrimental role in stroke outcome. Dramatic intracellular calcium elevation results from the hyper-activation of glutamate receptors and concurrent calcium release from intracellular storage [15]. Elevated intracellular calcium subsequently activates calcium-dependent enzymes, including DNases, proteases, and lipases, resulting in cell death under ischemic conditions [16]. However, clinical trials testing calcium blockers have failed to provide neuroprotection in patients after cerebral ischemia [17]. Previous studies have increasingly supported the idea that enhancing calcium also plays a neuroprotective role by triggering endogenous protective pathways, which may explain the failure of these clinical trials. CaMKK is a major kinase that is activated by intracellular calcium, and it targets multiple critical pathways for neuronal death/survival. Here, we have demonstrated that eliminating CaMKK β or CaMK IV exacerbates infarcts, edema formation and behavioral deficits. We have previously demonstrated the effects of CaMKK in maintaining the integrity of the BBB after stroke, which is particularly important because BBB disruption is one of the major contributing factors to edema formation and hemorrhagic transformation in cerebral ischemia [9,18]. The inhibitory effect of CaMKK on peripheral neutrophil activation and egress has been suggested as a possible mechanism for BBB protection [9]. Additionally, we provide evidence that CaMKK signaling may function to augment the transcription of neuroprotective genes such as BCL-2. Previous studies have suggested that deleting either CaMKK β or CaMK IV in males exacerbates the nuclear accumulation of histone deacetylase 4 (HDAC4) [9], which may in turn reduce the levels of the neuronal survival factors p-CREB and BCL-2 [19-21]. However, a direct link between CaMKK signaling and the regulation of HDAC4 translocation in females after stroke has not yet been established. Of note, endothelial cell, an important component of the neurovascular unit, may contribute to stroke outcome, particularly changes of BBB integrities after stroke. Interestingly, endothelial cells also express CaMKK [9]. Based on our data demonstrating a worse BBB in the KO after stroke, we speculate that inhibition of CaMKK signaling may enhance endothelia cell death and may contribute to greater HT observed in the KO mice. However, the role of CaMKK in endothelium under ischemic stress is still unclear and remains to be investigated.

Our data highlighted the importance of CaMKK signaling in protecting BBB in stroke. t-PA is currently the only FDA approved drug for stroke treatment but has a very narrow therapeutic window due to its risk of inducing HT. It would be very interesting in the future to study if targeting CaMKK signaling help reduce the risk of HT and extend the therapeutic window of t-PA. Indeed, bigger infarcts in KO mice may lead to worse BBB disruption and subsequently higher levels of hemoglobin in stroke brain. We therefore did a correlation analysis (data not shown) to see if higher amounts of hemoglobin is correlated to higher infarct volumes in both KO mice. No significant correlation was observed although there was a trend toward statistical significance in the CaMKK β KO mice (p = 0.11). We chose 6 hour time point to examine the changes of MMP activity and BBB protein levels et al., as at this time point, stroke infarct is not yet mature. Therefore changes in molecule signaling are more likely to be mechanistic than only a correlation with infarct size. Additionally, our previous published data suggested at this time point, there were significance changes of molecule signaling in males in CaMKK KO and CaMK IV KO mice after stroke [9]. In the current study we would like to see if the similar changes were occurring in females. We will examine the temporal profile of these molecule changes after stroke in our future studies.

In addition to its effects on BBB integrity and transcriptional activation, our data demonstrate that CaMKK signaling may induce neuroprotection through a reduction in post-stroke inflammation. The inflammatory response is increasingly recognized as a key contributor to stroke damage and is an attractive target for neuroprotective therapies because it takes place in a delayed manner. The CaMKK pathway regulates the activity of several transcription factors such as CREB, activator protein-1 (AP-1), myocyte enhancer factor-2 (MEF2), and members of the retinoid orphan receptor (ROR) family, which are all proteins that play pivotal roles in the immune response and inflammation by regulating processes such as T-cell development and cytokine secretion [14,22-25]. For example, CaMK IV stimulates ROR α-dependent transcriptional activation, which is responsive to changes in intracellular calcium in HaCaT cells [22]. ROR α has been suggested to function as a negative regulator of cytokines such as IL-6 and TNFα because the genetic deletion of ROR α greatly enhances the induction of these cytokines in mast cells and macrophages after LPS treatment [26,27]. Consistent with these earlier in vitro studies, we found that knockout of CaMKK β or CaMK IV increased TNFα and IL-6 production in mice after stroke. Therefore, the exacerbation of the inflammatory response seen in these KO mice may contribute to their worsened outcome after stroke. However, the mechanism by which CaMKK signaling reduces the inflammatory response after stroke and whether ROR α is involved in this process remains unclear. Of note, in the current study we only measured the inflammatory markers in peripheral blood after stroke. Although it is well established that peripheral inflammatory factors are able to infiltrate into the brain and exacerbate stroke outcome, our future studies will directly focus on the inflammatory response in the brain.

We used ovariectomized KO and WT control mice in this study primarily because these animals may be a more appropriate model for women at the highest risk for stroke, the majority of whom are postmenopausal [4]. Additionally, performing ovarietomy in female mice allows us to control levels of gonadal hormones therefore to reduce experimental variability that is associated with varying estrogen levels during estrus. We confirmed the loss of gonadal production of estrogen by showing a dramatic reduction in uterine weights after OVX. However, the direct effects of estrogen on the CaMKK signaling pathway in stroke remain unknown because we did not directly study stroke outcome in ovary-intact or estrogen-supplemented OVXed KO females.

Although CaMKK β has been shown to affect hippocampal memory formation in a sexually dimorphic manner [28], we found no overall difference between the sexes in the role of this pathway in response to stroke. In fact, deleting CaMKK or CaMK IV at the genetic level exacerbated the stroke outcome in both males [9] and females in a nonspecific manner. This suggests that CaMKK signaling plays a protective role for both male and female stroke victims. Discrepancies between this result and an earlier report that found sex-specific differences may result from differences in models or particularly in the brain regions targeted in each case. For example, the hippocampus is not directly affected by MCA occlusion, although it may be affected through secondary ischemia caused by stroke-induced brain edema formation. We did not specifically assess memory formation after stroke in females because the primary focus of our study was to identify whether the CaMKK pathway can exert neuroprotective properties in female animals; otherwise, targeting this pathway in females might fail to reduce injury.

## Conclusions

In summary, we have demonstrated that genetically inhibiting the CaMKK pathway is detrimental in the response of female mice to cerebral ischemia. Therefore, CaMKK signaling may play a neuroprotective role in both male [9] and female stroke victims. This result suggests that the CaMKK pathway may be a potential target for stroke therapy.

## Additional files

Additional file 1: Figure S1. Effect of CaMKK β and CaMK IV on hemispheric swelling after stroke. Swelling (%) = (Ipsilateral hemisphere- Contralateral hemisphere)/Contralateral hemisphere *100%. A, n = 7 WT/ n = 9 KO, B, n = 8 for each group; ^#^p < 0.05: versus WT controls. Data were showed as mean ± SEM. Additional file 2: Table S1. Ovxed CaMKK β/CaMK IV KO mice had increased neurological deficit scores after stroke.

## Abbreviations

CaMKK: Calcium/calmodulin-dependent protein kinase kinase
BBB: Blood brain barrier
p-CREB: Phosphorylated cAMP response element-binding protein
BCL-2: B-cell lymphoma 2
TNFα: Tumor necrosis factor alpha
IL-6: Interleukin 6
PARP: Poly-ADP-ribose polymerase
STAIR: Stroke Therapy Academic Industry Roundtable
AP-1: Activator protein-1
MEF2: Myocyte enhancer factor-2
ROR: Retinoid orphan receptor
