# Supplementary figures and images for: Genetic deletion of calcium/calmodulin-dependent protein kinase kinase β (CaMKK β) or CaMK IV exacerbates stroke outcomes in ovariectomized (OVXed) female mice
Source: BMC Neurosci. 2014 Oct 21;15:118. doi: 10.1186/s12868-014-0118-2 (PMC4207892; doi:10.1186/s12868-014-0118-2)

## Slide 1
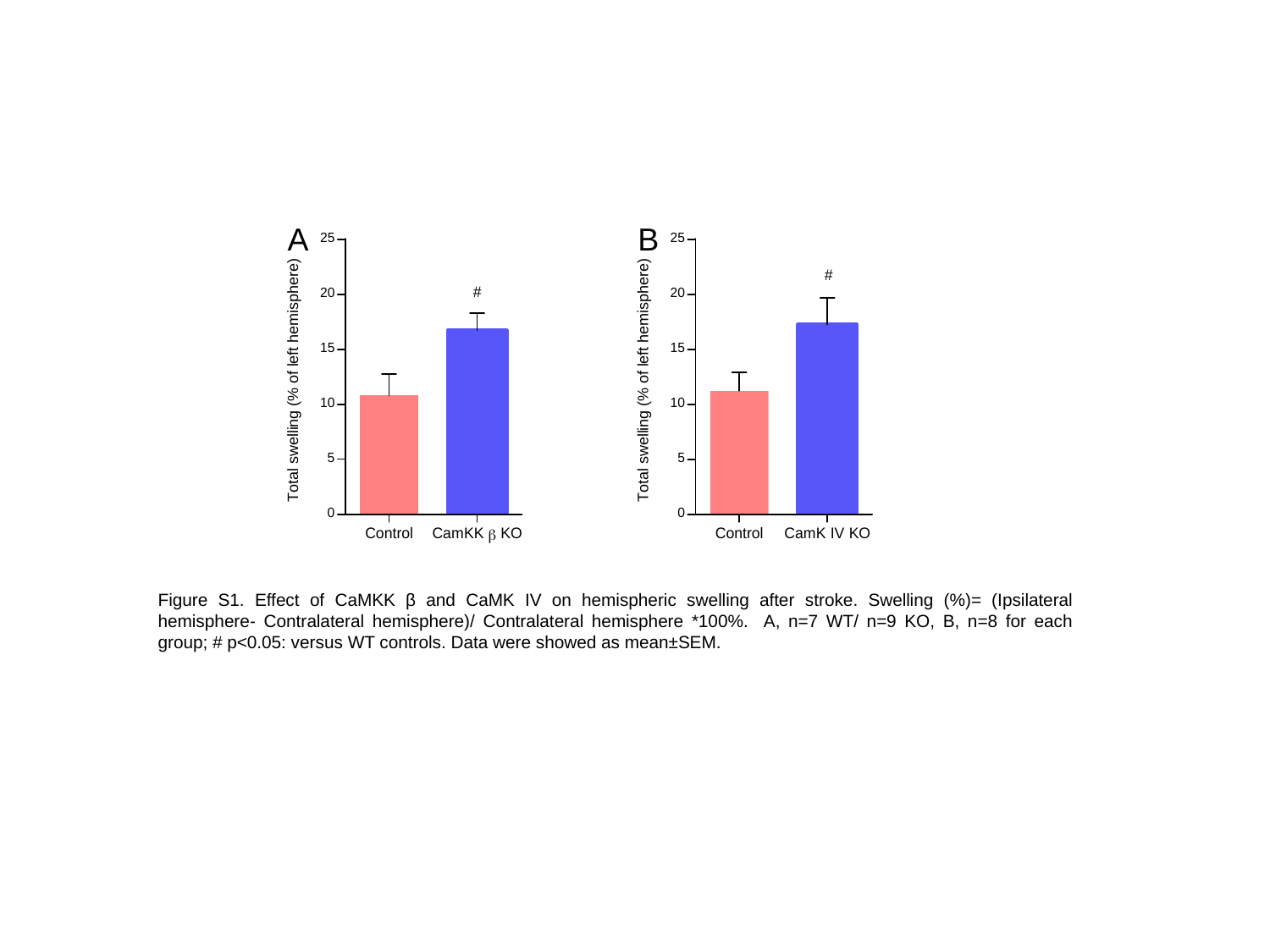

Supplement: Additional file 1: Figure S1. — Effect of CaMKK β and CaMK IV on hemispheric swelling after stroke. Swelling (%) = (Ipsilateral hemisphere- Contralateral hemisphere)/Contralateral hemisphere *100%. A, n = 7 WT/ n = 9 KO, B, n = 8 for each group; #p < 0.05: versus WT controls. Data were showed as mean ± SEM. [file 12868_2014_118_MOESM1_ESM.pptx]

## Slide 1
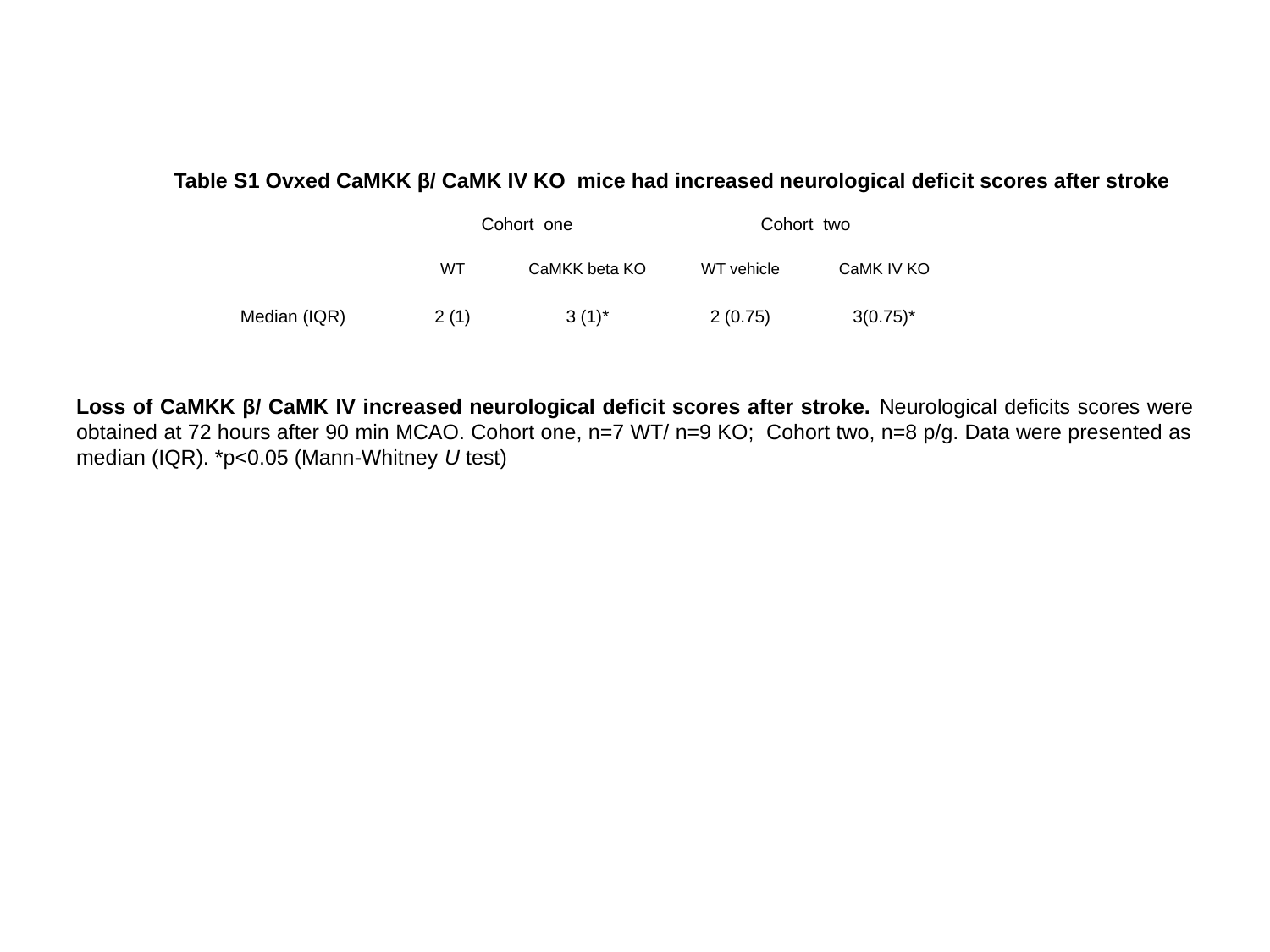

Supplement: Additional file 2: Table S1. — Ovxed CaMKK β/CaMK IV KO mice had increased neurological deficit scores after stroke. [file 12868_2014_118_MOESM2_ESM.pptx]
